# A disorder of surfactant metabolism without identified genetic mutations

**DOI:** 10.1186/s13052-015-0198-3

**Published:** 2015-11-25

**Authors:** Silvia Montella, Timothy J. Vece, Claire Langston, Paola Carrera, Lawrence M. Nogee, Aaron Hamvas, Angelo Manna, Mara Cervasio, Francesca Santamaria

**Affiliations:** Department of Translational Medical Sciences, Federico II University, Via Sergio Pansini, 5 – 80131 Naples, Italy; Department of Pediatrics, Baylor College of Medicine, Texas Children’s Hospital, Houston, TX USA; Department of Pathology, Baylor College of Medicine, Texas Children’s Hospital, Houston, TX USA; Division of Genetics and Cell Biology, IRCCS Ospedale San Raffaele, Milano, Italy; Department of Pediatrics, Division of Neonatology, Johns Hopkins University School of Medicine, Baltimore, MD USA; Edward Mallinckrodt Department of Pediatrics, Division of Newborn Medicine, Washington University School of Medicine, St. Louis, MO USA; Department of Pediatrics, Division of Neonatology, Ann and Robert H. Lurie Children’s Hospital of Chicago, Northwestern University Feinberg School of Medicine, Chicago, IL USA; Department of Advanced Biomedical Sciences, Anatomo-Pathology Unit, Federico II University, Naples, Italy

**Keywords:** Interstitial lung disease, Surfactant Biology and Pathophysiology, Genetic testing

## Abstract

**Background:**

Surfactant metabolism disorders may result in diffuse lung disease in children.

**Case presentation:**

We report a 3-years-old boy with dry cough, progressive hypoxemia, dyspnea and bilateral ground glass opacities at chest high-resolution computed tomography (HRCT) who had no variants in genes encoding surfactant proteins or transcription factors. Lung histology strongly suggested an abnormality of surfactant protein. A 7-month course of pulse intravenous high-dose methylprednisolone *plus* oral hydroxychloroquine and azithromycin led to gradual weaning from oxygen and oral steroids, and to improvement of cough and dyspnea. Over the follow-up period, hydroxychloroquine and azithromycin were not withdrawn as cough and dyspnea re-appeared at each attempt and disappeared at re-start. At 6 years of age chest HRCT still appeared unchanged, but clinical symptoms or signs were absent.

**Conclusions:**

In children suspected of inborn errors of pulmonary surfactant metabolism who do not have a recognized genetic mutation, lung biopsy with consistent histology may help physicians to address the definitive diagnosis.

## Background

Pediatric diffuse lung disease (DLD) is a heterogeneous group of uncommon disorders with impaired gas exchange and diffuse infiltrates at chest imaging [1].

Deletions of or mutations in genes encoding proteins important in surfactant production and function (SP-B, SP-C, and ABCA3), surfactant catabolism (GM-CSF receptor), or transcription factors important for surfactant production (TTF1) or lung development (Fox F1) may cause pediatric DLD [2]. The prognosis of surfactant disorders is heterogeneous, with high mortality in SP-B deficiency and variable severity in cases with mutations in *ABCA3* or SP-C [1, 3, 4]. Mutations of surfactant protein genes are detected in most patients [2]. However, 20–30 % of cases with histological pattern compatible with surfactant disorders have no identified mutation [1].

We report a young child in whom lung histology strongly suggested a surfactant disorder, but no variants conclusively classified as pathogenic in genes encoding surfactant proteins or transcription factors important for surfactant production were found.

## Case presentation 

The child was a full-term male, born to unrelated parents. There was no family history of lung disease. At birth he required no resuscitation. By age 3 years he developed fever with dry cough and dyspnea. Chest X-ray showed mild-to-moderate interstitial thickening, and IgM for *Mycoplasma pneumoniae* was detected. After 21 days of clarithromycin, fever disappeared. Respiratory symptoms persisted unchanged also after two-month course of inhaled steroids and montelukast. By age 3.6 years he was admitted to our hospital with persistent dry cough and dyspnea, and progressive hypoxemia. On examination, he had fine crackles and expiratory wheezes bilaterally. Transcutaneous oxygen saturation (SpO_2_) was 90 % at room air, but rose to 98 % at 4 liters/minute oxygen delivered by nasal cannula. The remainder examination also including neurologic assessment was normal. He could not perform spirometry.

**Fig. 1:**
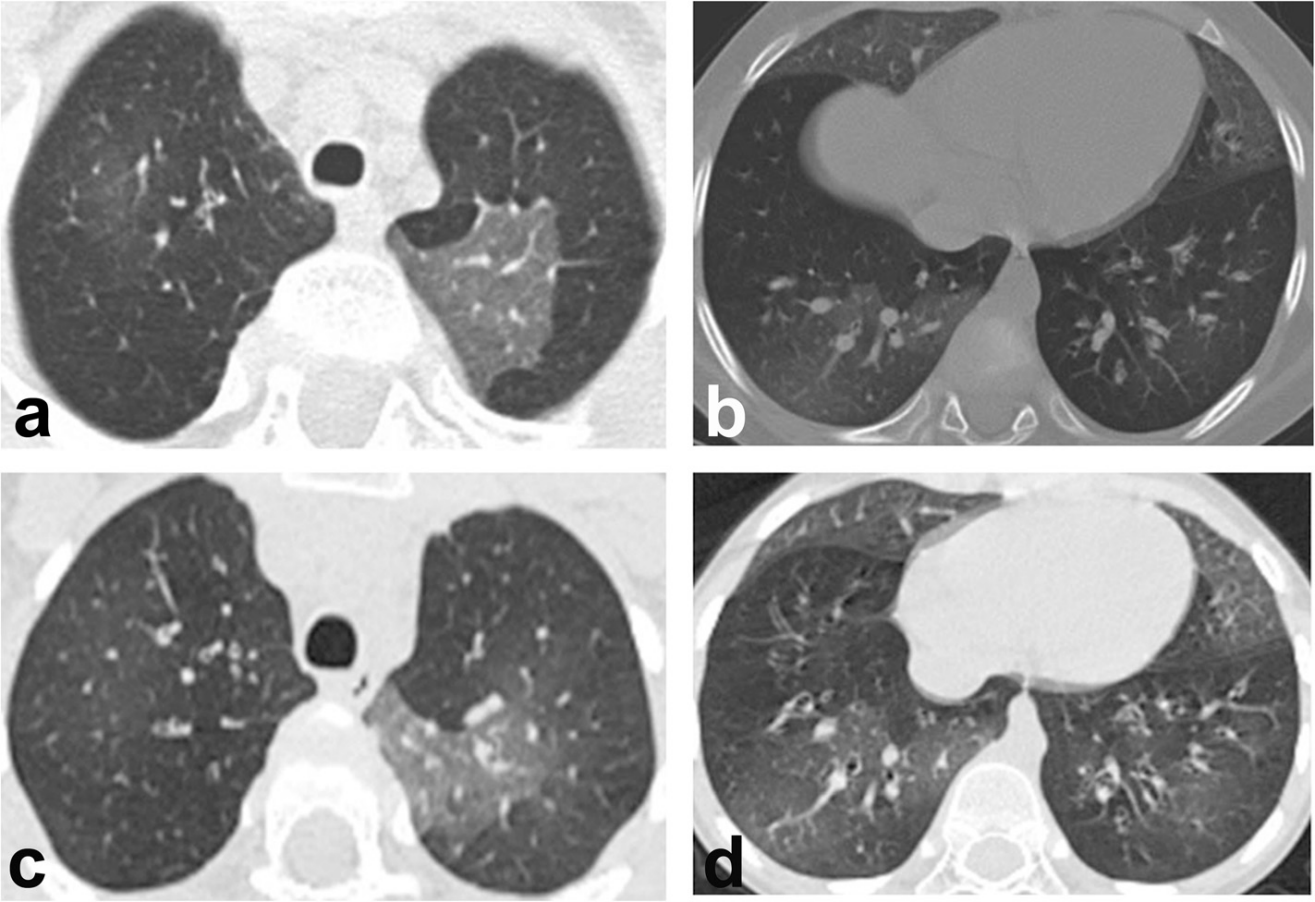
Baseline high-resolution computed tomography scans. Sharply defined areas of ground glass opacity more marked in the left upper lobe (**a**), in the middle lobe, lingula and right lower lobe (**b**). High-resolution computed tomography at the age of 6 years (after 3 years of oral hydroxychloroquine and azithromycin) showing unmodified ground glass attenuation in the same areas, indicating lung damage stabilization (**c** and **d**)

Structural cardiovascular abnormalities were excluded at echocardiography. Serological investigations ruled out *C. pneumoniae,* adenovirus, metapneumovirus, cytomegalovirus, and respiratory syncytial, influenza, parainfluenza, Epstein-Barr, and human immunodeficiency viruses. Airways anomalies were not evident at flexible bronchoscopy. We did not perform bronchoalveolar lavage because it has limited value in identifying the specific DLD or guiding therapy [1]. A 24 h pH-impedance monitoring was normal. The sweat test was negative and the genetic testing did not identify any causative mutation of cystic fibrosis. Ultrastructural analysis of nasal cilia was normal. Primary immune defects were ruled out and serological testing for connective tissue disorders were negative. Thyroid function was normal. Chest high-resolution computed tomography (HRCT) revealed multiple bilateral ground glass opacities (Fig. 1a and b). A direct sequencing protocol was applied to the coding regions and exon/intron junctions of *SFTPB*, *SFTPC*, *ABCA3*, *NKX2.1*, and *NKX2.5* genes. Promoter and regulatory regions were not analyzed because at present no mutation has been described in these sites. Molecular analysis in these genes did not reveal a pathogenic variant in the patient. However, the IVS4+39 C>T heterozygous rare variant (rs79253830) was identified in *SFTPC* by Sanger direct sequencing. The child was also heterozygous for the known *ABCA3* single nucleotide polymorphisms rs170447, rs313908, and rs149532.

**Fig. 2:**
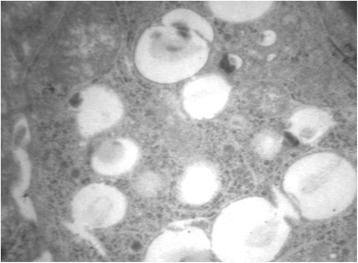
Type II pneumocyte cytoplasm. Numerous small structures containing irregular and poorly staining material at electron microscopy

A thoracoscopic lung biopsy was performed. Histology showed widened alveoli with thickened alveolar septa infiltrated mainly by fibroblasts and lymphocytes, hyperplasia of type-II pneumocytes, and intra-alveolar accumulation of finely granular periodic acid Schiff-positive material and several cholesterol crystals, suggesting pulmonary alveolar proteinosis and nonspecific interstitial pneumonia. At electron microscopy, alveolar type II cells lacked mature cytoplasmic lamellar bodies, while other organelles appeared normal, which was consistent with surfactant protein abnormality. In particular, type II cell cytoplasm showed numerous small structures containing irregular and poorly staining material (Fig. 2).

Pulse intravenous high-dose methylprednisolone (30 mg/kg once daily for three successive days each month), oral hydroxychloroquine (10 mg/kg once daily) and azithromycin (10 mg/kg once daily for three consecutive days/week) were started. Prior to treatment, the patient underwent a six minute walking test that showed a walk distance of 381 (72 % predicted) meters and a minimum SpO_2_ during the walk of 81 %. Moreover, he spent 89.4 % of the walk time with SpO_2_ <90 %. The combined therapy led to gradual weaning from oxygen and improvement of cough and dyspnea over seven months. Oxygen and steroids were gradually withdrawn, but oral hydroxychloroquine and azithromycin continued. Six months after steroids and oxygen were stopped, a substantial improvement of exercise tolerance *versus* pre-treatment test was observed as walk distance was 441 meters (83 % predicted), with minimum SpO_2_ during the walk of 85 % and 45.5 % of the walk time spent with SpO_2_ <90 %.

Over the follow-up, we tried to withdraw hydroxychloroquine and azithromycin, but cough and dyspnea re-appeared at each attempt, and rapidly disappeared at re-start. Currently, the subject is 6 years-old, adherence to treatment is satisfactory, with no side effects. Repeated daily SpO_2_ showed values not lower than 98 % at room air, with median overnight values of 96 % (min 74 %, max 99 %). He is still unable to perform spirometry. Chest HRCT is unchanged (Fig. 1c and d).

## Discussion

In this child with no mutations in genes important for surfactant function and metabolism, lung biopsy suggested surfactant dysfunction, but with some unusual features including the absence of lamellar bodies. The significance of this finding is unclear. However, considering the normal ultrastructure of other cellular and nuclear membranes, it is thought to be a real finding, unrelated to biopsy processing or handling. Given the patient’s phenotype, this pattern is consistent with a disorder of surfactant metabolism.

For infants or young children with clinical, imaging, and/or histological features suggestive of DLD, testing for genetic abnormalities associated with surfactant dysfunction and/or NKX2.1 is recommended [2]. Although positive results may obviate the need for lung biopsy, individuals with unresolved disease in whom mutations are not found require tissue sampling for achieving a definitive diagnosis and guiding further diagnostic and therapeutic choices [1].

Indeed, the recent scheme for pediatric DLD includes conditions with histology consistent with surfactant dysfunction disorder without a yet recognized genetic mutation [1, 5]. Actually, the sensitivity of genetic testing for surfactant dysfunction disorders was not formally evaluated and, although the frequency of false-negative results cannot be precisely estimated, it has been assumed to be low [1]. In particular, current analytical methods cannot detect all mutations that cause surfactant disorders (i.e., large rearrangements identified by conventional Sanger sequencing). Moreover, functionally significant variants may be present in untranslated regions that are not examined through routine clinical sequencing or in other genes not yet associated to the disease [6]. In our case, standard sequencing methodologies did not detect mutations in *SFTPB*, *ABCA3*, and *NKX2* genes, but only identified the *SFTPC* IVS4 + 39C > T rare variant located 21 nucleotides downstream to the termination codon, in the 3′UTR. Unfortunately, we could not perform RNA analysis of the lung tissue and, although *in silico* algorithms did not predict an effect on RNA splicing, we cannot definitely exclude a possible pathogenic role of the IVS4+39 C>T variant. Furthermore, we cannot exclude the presence of a second unidentified mutation on the other allele.

As the child was heterozygous for known *ABCA3* single nucleotide polymorphisms, homozygosity by descent for the same rare *ABCA3* allele is improbable. Thus, variants in untranslated regions of or deletions in *ABCA3* unlikely explain the child’s illness, as such variants are a small minority of reported disease-causing *ABCA3* alleles and would have to be present on two different *ABCA3* alleles. Finally, given the complexity of surfactant synthesis, secretion, and catabolism, there are likely to be as yet unidentified disorders of other molecules in this pathway that, when mutated, result in a similar histological phenotype.

Treatment of SP-C and ABCA3 abnormalities derives from anecdotal experience. The variable clinical course of pulmonary disease, combined with the lack of prospective randomized controlled trials, makes it difficult to uniform treatment strategies [2]. Treatment is based on the concept that suppressing inflammation is supposed to prevent the progression to pulmonary fibrosis. Among the anti-inflammatory agents, steroids are the preferred choice. As glucocorticoids increase ABCA3 expression in vitro [7], there is a rationale for intravenous pulse steroids use in ABCA3 deficiency. Given the limited evidence of a beneficial effect on clinical outcomes and the well known side effects of immunosuppressive medications, whether or not to initiate a trial of immunosuppressive therapy must be decided on an individual basis. If steroids are given, patients should be closely monitored for side effects, including periodic measurement of weight and height, glucose homeostasis, bone density, and ophthalmologic screening. Hydroxychloroquine, an anti-malarial drug supposed to have immunological effects [8], has also been often used for treating DLD in children. If this drug is administered, eye exams must be routinely performed to look for retinal toxicity, although this complication is extremely rare in children. Likewise, azithromycin has been used for its anti inflammatory effects [1, 9]. As surfactant disorders are incurable, supportive care is important for maintaining optimal health status. This includes oxygen administration, if required, and adequate nutritional support as well as interventions to prevent infections such as pneumococcal vaccine, annual influenza vaccination and routine childhood immunizations. Lung transplantation is an ultimate option if progressive clinical deterioration unresponsive to medical therapy develops [3, 4, 9]. In the current case, the combined treatment of pulse methylprednisolone and hydroxychloroquine *plus* azithromycin resulted in progressive clinical resolution and lung damage stabilization. Finally, a close follow-up is crucial to monitor the progression of disease.

## Conclusions

Our report confirms that in children with suspected surfactant dysfunction, genetic testing is strongly recommended because it can provide a definitive diagnosis, may obviate unnecessary procedures and interventions, and potentially provide important prognostic information for families and physicians [1]. Nevertheless, physicians should be aware of the possibility of lung histology consistent with surfactant disorder without a yet recognized genetic mutation.

## Ethical approval

All procedures performed in this publication are in accordance with the ethical standards of the institutional and/or national research committee and with the 1964 Helsinki declaration and its later amendments or comparable ethical standards.

## Consent

Written informed consent was obtained from the parents of the patient for publication of this Case report and any accompanying images. A copy of the written consent is available for review by the Editor-in-Chief of this journal.

## Abbreviations

DLD: Diffuse lung disease
HRCT: High-resolution computed tomography
