# Erratum to: A disorder of surfactant metabolism without identified genetic mutations

**DOI:** 10.1186/s13052-015-0205-8

**Published:** 2015-12-16

**Authors:** Silvia Montella, Timothy J. Vece, Claire Langston, Paola Carrera, Lawrence M. Nogee, Aaron Hamvas, Angelo Manna, Mariarosaria Cervasio, Francesca Santamaria

**Affiliations:** Department of Translational Medical Sciences, Federico II University, Via Sergio Pansini, Naples, 5 – 80131 Italy; Department of Pediatrics, Baylor College of Medicine, Texas Children’s Hospital, Houston, TX USA; Department of Pathology, Baylor College of Medicine, Texas Children’s Hospital, Houston, TX USA; Division of Genetics and Cell Biology, IRCCS Ospedale San Raffaele, Milano, Italy; Division of Neonatology, Department of Pediatrics, Johns Hopkins University School of Medicine, Baltimore, MD USA; Division of Newborn Medicine, Edward Mallinckrodt Department of Pediatrics, Washington University School of Medicine, St. Louis, MO USA; Division of Neonatology, Department of Pediatrics, Ann and Robert H. Lurie Children’s Hospital of Chicago, Northwestern University Feinberg School of Medicine, Chicago, IL USA; Department of Advanced Biomedical Sciences, Anatomo-Pathology Unit, Federico II University, Naples, Italy

Unfortunately, the original version of this article [1] contained an error. The name of one of the authors was included incorrectly and it read “Mara Cervasio” instead of “Mariarosaria Cervasio”.

The name has been updated in the original article and is also correctly included in full in this erratum.
